# Vertical transportation systems embedded on shuffled frog leaping algorithm for manufacturing optimisation problems in industries

**DOI:** 10.1186/s40064-016-2449-1

**Published:** 2016-06-22

**Authors:** Pasura Aungkulanon, Pongchanun Luangpaiboon

**Affiliations:** Industrial Statistics and Operational Research Unit (ISO-RU), Department of Industrial Engineering, Faculty of Engineering, Thammasat University, Pathumthani, 12120 Thailand

**Keywords:** Vertical transportation system, Shuffled frog leaping algorithm, Single pass turning, Multi-pass turning

## Abstract

Response surface methods via the first or second order models are important in manufacturing processes. This study, however, proposes different structured mechanisms of the vertical transportation systems or VTS embedded on a shuffled frog leaping-based approach. There are three VTS scenarios, a motion reaching a normal operating velocity, and both reaching and not reaching transitional motion. These variants were performed to simultaneously inspect multiple responses affected by machining parameters in multi-pass turning processes. The numerical results of two machining optimisation problems demonstrated the high performance measures of the proposed methods, when compared to other optimisation algorithms for an actual deep cut design.

## Background

As a result of continuous changes in business environment and uncertainty of demand and production processes, manufacturing industries are having to develop and evolve rapidly. Therefore, in the future, the production system should be flexible and able to respond to factor alterations quickly. The system should create high quality products, support small production volumes and meet all needs of customer specifications. Modern production systems consist of high-performance technology and equipment, which increase production capacity. However, at the same time, the system operations become more sophisticated and complex. Manufacturing processes involve inputs from various departments including management, production, finance, marketing, and engineering etc. Hence, the created production system should be able to allow relevant departments to influence the manufacturing operations conveniently and effectively. The production system should consist of the following three components of inputs including manpower, raw materials, machines, energy, money and information; processes including preparation of materials, assembly of all components into various shapes as well as packaging for distribution and outputs including products or outputs in the form of goods or services. Turning is a basic process that is commonly used in various industries. The operation involves rotating the work-piece while moving a cutting tool linearly toward the work-piece to improve the work-piece. Turning can cut or decrease the size of a work-piece by removing external surface with a cutting tool positioned vertically to its rotating axis. Types of turning methods include facing, straight turning, thread turning, boring, necking and parting. Lathe machines can be classified as manually controlled or automatically controlled.

Recently, some meta-heuristic methods can provide better solutions for various manufacturing optimisation problems. The objectives of multi-pass turning and single-pass operation optimisations are significantly different. Multi-pass turning operations finally finish the surface to achieve the desired condition. The single-pass operation intends to gain the highest possible material removal rate (MRR) under various machining performance measures. The minimum cost, maximum MRRs, longer tool life, a lower cutting force, and better surface roughness are affected by the actual combination of cutting parameters. Both objectives of minimising total production cost and minimising machining time are considered quite often in related mathematical models in literatures. There are various cutting constraints considered in machining operations. In turning operations, either single or multiple passes are used for a cutting process. For economic reasons, multiple pass turning is preferable over the single pass turning in almost industries. Machining models consist of some parameters such as machining time, metal removal rate, tool waste and tool life. Several researchers have investigated the optimisation of cutting parameters in turning operations with a variety of models. The machining parameters have been determined by various methods. They consist of conventional modes of deterministic, probabilistic or dynamic programming method (Ermer and Patel 1974). However, these traditional optimisation methods may not be robust due to various complications of multiple constraints and passes. Their solutions are not ideal for solving machining optimisation problems because they tend to obtain a local optimal solution. Thus, meta-heuristic algorithms and their hybridisations have developed for solving machining problems due to their power in searching for a global optimum.

The meta-heuristic optimisation algorithms proved that they seem to be better than the traditional methods in many applications. Several interesting researches based on machining optimisation problems have been reported in the past, many claiming improved algorithms performance. Yildiz and Ozturk (2006) developed the Taguchi method to determine the proper levels of controllable design variables. Two multi-pass turning problems were optimised by the genetic algorithm (GA) to get the new settings of design variables. The results found by the hybrid robust genetic algorithm (HRGA) were better than those of scatter search, GA and simulated annealing and hooke–jeeves pattern search (SA/HJPS) for turning operations. From some recent empirical and theoretical reports on collective behaviors based on a topological interaction, the GA can be applied to the swarm dynamics (Shang and Bouffanais 2014). Wang (2007) studied an ant colony optimisation method for determining the machining parameters in a multi-pass turning operation model. The ant colony method was better than other optimisation techniques developed by other researchers. Their conclusion showed that the optimal solution as found by Vijayakumar et al. (2003) was not valid. Vijayakumar and Kumudinidevi (2007) proposed a new optimisation technique based on the ant colony algorithm for solving multi-pass turning optimisation problems. Yıldız (Zarei et al. 2009) developed a hybrid method by combining an immune algorithm with a hill climbing local search algorithm for solving optimisation problem. The hybrid algorithm combined the exploration speed of the immune algorithm with the powerful ability to avoid being trapped in local minima of the hill climbing. The results demonstrated the proposed hybrid method significantly outperformed, when compared to other techniques in terms of solution quality and convergence rates. Two similar studies by Chen and Chen (2010) and Onwubolu and Kumalo (2001) compared the effectiveness of the GA with several solution algorithms in solving machining operating problems. By using the problem of Chen and Tsai (1996), they concluded that the GA was significantly better than a simulated annealing. Yildiz (Yildiz 2013d) conducted a study to compare three meta-heuristic algorithms of an artificial bee colony (ABC), a particle swarm optimisation (PSO), and a simulated annealing (SA) for optimising parameters on multi-pass milling processes.

Yildiz (2012) showed the superiority of the hybrid approach over many other techniques. They consisted of an artificial bee colony algorithm, a differential evolution algorithm, a hybrid particle swarm optimisation algorithm, a hybrid artificial immune-hill climbing algorithm, a hybrid Taguchi-harmony search algorithm, a hybrid robust genetic algorithm, a scatter search algorithm, a genetic algorithm and an improved simulated annealing algorithm. The performance was measured via a convergence speed or the required number of function evaluations. The hybrid of the differential evolution algorithm with a receptor editing property of an immune system (DERE) was more effective for optimising machining parameters, when compared to other approaches. This evidence has been claimed to be representative of the state-of-the-art in evolutionary optimisation literatures in machining optimisations. Yusup et al. (2012) used a GA to optimise process parameters on the largest machining operations of a multi-pass turning. In terms of machining performance, surface roughness was mostly studied with meta-heuristic algorithms. Hybrid evolutionary optimisation algorithms could solve the problem with a fast convergence and robustness for finding the global minimum at the same design points. Dep and Datta (2011) used an evolutionary multi-objective optimisation (EMO) with a suitable local search procedure to optimise the machining parameters in turning operations. These parameters were cutting speed, feed and depth of cut. The study concluded the EMO solutions were computationally faster than the original EMO results. Belloufi et al. (2012) proposed a new hybrid algorithm with genetic and sequential quadratic programming procedures for a resolution of cutting conditions. The resolution of a multi-pass turning optimisation case was to minimise the production cost under a set of machining constraints. The proposed hybrid algorithm was better than other techniques carried out by different researchers.

In a study by Rao and Kalyankar (2013) compared a teaching learning-based optimisation algorithm with various previously attempted algorithms such as a simulated annealing, a genetic algorithm, an ant colony algorithm, and a particle swarm optimisation. The teaching–learning-based optimisation algorithm was effective, when compared to other algorithms. Lu et al. (2013) presented a new approach to optimise the cutting pass sequences and machining parameters in turning operations with practical constraints. A hybrid solver was a hybrid of a genetic algorithm and a sequential quadratic programming technique. Belloufi et al. (2014) used a firefly algorithm (FA) and a hybrid of a genetic algorithm and a sequential quadratic programming (GA-SQP) for the machining parameters in a multi-pass turning operation model. Mellal and Williams (Mellal and Williams 2015) developed and compared the cuckoo optimisation algorithm (COA) with a wide range of optimisation algorithms. The COA required a lower number of function evaluations, improved the convergence rate, and showed its ability to handle different constraint forms. Chauhan et al. (2015) used Totally Disturbed Particle Swarm Optimisation (TDPSO) to optimise machining conditions during multi-pass turning operations with various constraints. They concluded that the TDPSO was efficient for dealing with cutting parameters optimisation in multi-pass turning operations. However, the complexity of machine parameter optimisation for economic machining problems still existed.

Recently, there have been a few researches reporting results of the application of the shuffled frog leaping algorithm (SFLA) to multi-objective manufacturing optimisation problems in industries. The original SFLA is easy to apply and has performed well on various engineering problems. Revisions are still possible to further explore its potential framework. In this new paper, variants of hybrid meta-heuristics algorithms based on the SFLA are introduced for determining the manufacturing optimisation problems. In order to improve the SFLA performance on complex optimisation problems, we apply various evolutionary elements, which are involved vertical transportation systems (VTS). Instead of applying the worst frog by its normal procedures as exemplars, mechanisms from a motion reaching a normal operating velocity, and both reaching and not reaching transitional motion can potentially be used as the exemplars to guide the frog with the better leaping direction. To further improve the search ability of the SFLA, variants of the frog leaping step size from the VTS are adjusted by performing the designed experiments. Effectiveness of all variants is shown by comparing the performance of a family of turning processes as reported in the literatures. The two machining problems deal with the design of single and multi-pass turning processes. The rest of the paper is organised as follows: next section discusses the “Vertical transportation system”. “Machining problems” section illustrates the details and mathematical models of the single and multi-pass processes. “Computational results and analyses” section explains “Shuffled frog leaping algorithm”. “Computational results and analyses” section provides the results and discussions including the related research of harmony and shuffled frog leaping algorithms on fundamental machining problems. Then, a summary, conclusions and further work are outlined in “Conclusion and future work” section.

## Vertical transportation systems

**Fig. 1:**
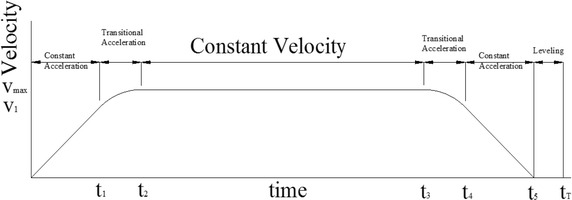
Pattern and order of an elevator movement

For high buildings, an elevator or lift is highly important to efficiently move people or goods between floors of a building. During elevator operation there are various influential parameters such as constant acceleration, transitional acceleration, constant velocity, transitional deceleration, constant deceleration, and leveling. By law, at least one firefighting elevator with the capacity to stop at every floor is required in all buildings. Also the continuous moving period of a firefighting elevator between the lowest and the top floor must not exceed 1 min (Klote 1993). The appropriate movement of an elevator has pattern and order after using the maximum high speed as shown in Fig. 1. This pattern can be used to calculate traveling time. The move starts by a constant acceleration. Then, when the transitional acceleration is reduced toward zero, the elevator moves with a constant speed and zero acceleration. Next, another transitional move happens, when the acceleration is increased from zero to the last constant step until the elevator stops. Lastly, the floor of the elevator adjusts to the building floor, which is called leveling. From the pattern and order of an elevator, there are some important design parameters. *v*_1_ is the velocity at the start of the transitional acceleration state. It is normally equal to 60 % of the maximum velocity *v*_*max*_, which compromises motion control and energy consumption for expected running time of an elevator. Based on elevator group control system, this level also improves traffic efficiency, reduces the chance of a long waiting time and the average time from when a passenger arrives at the hall until when the passenger boards an assigned car, and eases passenger frustration, especially during the morning up peak. It is simultaneously achieved via various criteria of performance, earth conscious, technology, intelligence and flexibility (Strakosch and Caporale 2010). Note that *t*_1_ is constant acceleration time, *t*_2_ is time to constant velocity, *t*_3_ is time at the end of constant velocity to start transitional acceleration, *t*_4_ is time to start of constant acceleration going down, *t*_5_ is time to finish of constant acceleration, when velocity equals zero, and *t*_*h*_ is time to leveling. An analysis of time and distance according to the movement of the elevator has three scenarios as follows. The first scenario is a motion reaching a normal operating velocity. The second and third cases are of motion reaching and not reaching transitional accelerations, respectively.

A calculation for the first scenario starts, when an elevator is stationary or speed is zero. The elevator then moves to increase speed with a constant acceleration (*a*) until reaching *v*_1_ at *t*_1_ in Fig. 1. The time spent (*t*_1_) to this stage can be determined by the following equation (Eq. 1). This vertical move travels a distance of *s*_1_ as shown in Eq. 2.

1 $$t_{1} = \frac{{v_{1} }}{a}$$

2 $$s_{1} = \frac{{v_{1}^{2} }}{2a}$$

For the transitional acceleration, the time spent (*t*_2_ − *t*_1_) is approximated by Eq. 3. The speed in this transitional period increases while the acceleration decreases to zero. A more accurate formula to calculate *t*_2_ may be unnecessary because the transitional deceleration period is very short, when compared to the whole movement of an elevator. During this period, the lift moves a distance (*s*_2_ − *s*_1_) as shown in Eq. 4. For one trip, the time spent before adjusting to the last floor is approximated by Eq. 5 and *s*_*t*_ is the distance for one trip. The total time spent (*t*_*T*_) including a leveling adjustment period (*t*_*h*_) is shown in Eq. 6. This adjustment time is normally 0.5 s.

3 $$t_{2} = \frac{{\left( {V_{max}^{2} - V_{1}^{2} } \right)}}{{2v_{1} a}} + t_{1}$$

4 $$S_{2} = \left( {\frac{1}{3a}} \right)\left( {\frac{{V_{max}^{3} }}{{V_{1} }} - V_{1}^{2} } \right) + S_{1}$$

5 $$t_{5} = 2t_{2} + \left( {\frac{{S_{T} - 2S_{2} }}{{V_{max} }}} \right)$$

6 $$t_{T} = t_{5} + t_{h}$$

**Fig. 2:**
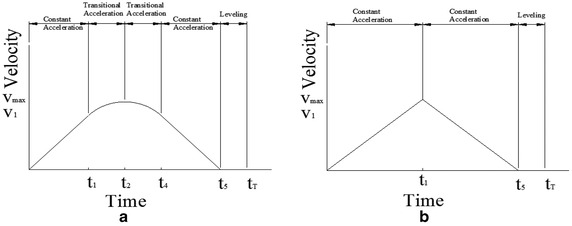
An elevator movement not reaching an ending point of transitional acceleration (**a**) and a motion not reaching a transitional acceleration (**b**)

In the following scenario shown in Fig. 2a, the elevator movement does not reach an ending point of transitional acceleration. The acceleration is not reduced to zero so there is no constant speed period. A calculation of *t*_1_ and *S*_1_ with a constant acceleration will be the same as the first case. The next period is a transitional acceleration period in which its speed does not reach a constant speed. The calculation follows Eq. 7. For an analysis of formula accuracy, the value of *V*_2_ at *t*_2_ in the second case should be similar to the first case. The period until the end of the transitional acceleration (*t*_2_) is shown in Eq. 8. The total time (*t*_*T*_) spent in one trip is as follows in Eq. 9.

7 $$V_{2} = \left[ {V_{1}^{3} + 3aV_{1} \left( {\frac{{S_{T} }}{2} - S_{1} } \right)} \right]^{1/3}$$

8 $$t_{2} = \frac{{\left( {V_{2}^{2} - V_{1}^{2} } \right)}}{{2aV_{1} }} + t_{1}$$

9 $$t_{T} = 2t_{2} + t_{h}$$

In a scenario of a motion not reaching a transitional acceleration as shown in Fig. 2b, the elevator does not move at a constant velocity resulting in bumping as deceleration starts. In a high building, this motion should not be allowed in a high speed elevator. For travel between adjacent floors and stop, the calculation of travelling time for one trip is as Eq. 10.

10 $$t_{T} = 2\sqrt {\frac{{S_{T} }}{a} + t_{h} }$$

These three variants are embedded on a shuffled frog leaping algorithm (SFLA). The basic SFLA was originally introduced by Eusuff and Lansey (2003) for a pipe network expansion optimisation. The SFLA separated a population into several memeplexes and then improved each memeplex in an evolutionary process. Various modifications have been proposed by different researchers to overcome the weaknesses of basic SFLA. Zhu and Zhang (2014) improved the original SFLA by allowing all frogs to take part in a memetic evolution and adding the self-variation behavior to the frog. It aimed to determine component pick-and-place sequences of a gantry multi-head component surface mounting machine. Earlier Elbeltagi et al. (2007) developed a new search via an acceleration parameter into the formulation of the original SFLA to create a modified form of the algorithm for two benchmark test problems including two discrete optimisation project management problems. Zhang et al. (2012) modified the basic SFLA by adding the basic ideas of an artificial fish (AF) algorithm for a cognitive radio system (CRS). They found the hybrid method provided better global convergence and less possibility to get trapped in local optimum. Roy (2011) introduced a hybrid solution method involving modified shuffled frog leaping algorithm (MSFLA) with a genetic algorithm (GA). It aimed at solving an economic load dispatch problem of generating units with valve point effects. Jadidoleslam and Ebrahimi (2015) developed a modified shuffled frog leaping algorithm (MSFLA) to solve a reliability-constrained generation expansion planning (GEP) problem. The new frog leaping rule of MSFLA was associated with a new strategy for frog distribution into memeplexes. The benefits of an integer encoding, a mapping procedure and a penalty factor approach were implemented to increase the efficiency of the proposed method, which aimed to improve the local exploration and performance of SFLA. Bhattacharjee and Sarmah (2014) modified a discrete shuffled frog leaping algorithm (MDSFL) to solve knapsack problems. The proposed algorithm included two important operations of the local search of the particle swarm optimisation technique and the competitiveness mixing of information of the shuffled complex evolution technique.

Yammani (2011) focused on an optimisation of weighting factors to balance the cost and the loss factors. An aim was to help build up desired objectives with a maximum potential benefit by the SFLA. Niknam et al. (2011) proposed an efficient multi-objective modified shuffled frog leaping algorithm (MMSFLA) for solving the multi-objective distribution feeder reconfiguration (MDFR) problem. Sharma et al. (2015) introduced a modified version of a shuffled frog leaping algorithm. A geometric centroid mutation was used to enhance the convergence rate. The proposal was implemented on five benchmark and car side impact problems. Simulated results illustrated the efficacy of the proposal in terms of convergence speed and mean value. Luo and Chen (2014) proposed a novel hybrid shuffled frog leaping algorithm (HSFLA) for a vehicle routing problem with time windows (VRPTW) with two strategies of a modified improvement procedure and a new memeplex construction. This approach was estimated and compared with other state-of-the-art heuristics using Solomon and Cordeau VRPTW test sets and showed the proposed algorithm was very effective for handling VRPTW. Kumar and Kumar (2014) proposed a shuffled frog leaping algorithm for an optimal market bidding strategy problem. The proposed method enhanced the short comings of selecting operators and premature convergence of a genetic algorithm (GA) and a particle swarm optimisation methods. Li et al. (2012) proposed a hybrid shuffled frog leaping algorithm (HSFLA) with a designed crossover operator for solving the multi-objective flexible job shop scheduling problem. Guo et al. (2015) proposed an improved shuffled frog leaping algorithm (SFLA) for the combinatorial optimisation problem of an assembly sequence planning (ASP). Under a remote handling maintenance in radioactive environment the improved SFLA was compared with the SFLA, genetic algorithm, particle swarm optimisation, and adaptive mutation particle swarm optimisation in terms of efficiency and capability of locating the best global assembly sequence. From experimental results the proposed algorithm exhibited an outstanding performance in solving the ASP problem. The application of the proposed algorithm also increased the level of the ASP in a radioactive environment.

The SFLA starts its sequential procedures by creating virtual frogs, which represent solutions or chromosomes for the GA. An optimisation process begins to determine the fittest virtual frog or solution. Then each of *m* memeplexes improves an optimised value of the frog with the smallest value. Each memeplex consists of *n* frogs. Therefore, the total population of frogs (*P*) in the memeplexes is equal to *m* multiplied by *n* (*P* = *m* * *n*). For an allocation method, the solution (frog) having the best fitness is arranged according to descending fitness. This best solution is assigned to the first memeplex. At the same time, the solution having second best fitness (frog 2) is assigned to the second memeplex. This is repeated until the *m*th frog or solution with the worst fitness is allocated into the *m*th memeplex or last memeplex. The *m* + 1 frog is then assigned to the first memeplex and so on, until all the frogs are allocated. In each memeplex, the best and worst fitness solutions are determined and set as *X*_*b*_ and *X*_*w*_, respectively. The solution having the best fitness in the global groups is defined as *X*_*g*_.

In an attempt to improve the worst fitness frog, total number of iterations of an evolution is determined. After these iterations, if the optimised value of the frog is still unimproved to reach the best frog (*X*_*g*_), the worst frog is eliminated and replaced by a new frog. The calculations of the frog leaping step size of the *i*th frog or *D*_*i*_, changing in the *i*th frog position based on the best (*X*_*b*_) and worst (*X*_*w*_) frogs, and the new position of the worst frog (*X*_*w*_), within the ranges of −*D*_*MIN*_ and $D_{MAX}$, are as follows in Eqs. 11 and 12, where *Rand*() is a random number in the range of [0, 1].

11 $$D_{i} = Rand() \times \left( {X_{b} - X_{w} } \right)$$

12 $$X_{w} = Current\,Position\,of\, X_{w} + D_{i}$$

In summary, there are eight SFLA optimisation procedures. For the first step, the parameters of the number of iterations in a memeplex and population of frogs are defined. The second is to generate an initial population of frogs using a randomisation. Steps 3 and 4 are to calculate the fitness value of each frog and arrange the frogs according to their descending fitness values. The fifth step, is an allocation of frogs into sub-groups or memeplexes based on the fourth step. The frog having the best fitness is assigned to the first memeplex. At the same time, the solution having the second best fitness is assigned to the second memeplex. This process is repeated until an allocation of all frogs is completed. Step 6 is to improve the frog with the worst fitness in each memeplex and test their fitness again. If the optimised value of the frog is still unimproved, the frog will be eliminated. A selection of the frog with the best fitness in each memeplex is done in the seventh step. A comparison is also made to determine the frog having best fitness in the population of the first iteration. Finally, the process is repeated according to the prescribed number of iterations. In the evolutionary process of the frog group, poor frogs affected by good frogs convert to be more robust to obtain more food. From a frog leaping rule (Step 6) an improvement process assists the algorithm search for better solutions. In fine-tuning of optimised solution vectors, the SFLA procedures can be useful in adjusting a convergence rate to an optimum. Therefore, new improvement processes of fine-tuning are of interest. The SFLA uses a selected position of the worst solution to be an improvement choice by randomly selecting an interval from the best to the worst solutions.

In the SFLA, all improvement generators of the worst solution cannot be changed during new generations. The weakness of the SFLA occurs, when it is at the high number of iterations. In some cases, it is impossible to provide a larger interval between the best and the worst solutions or to overcome getting stuck at the local optimum. Thus this brings the difficulty in finding the better value for the current worst solution. The range of the global best and best solutions may also decrease algorithm performances. This significantly increases the needed iterations without any improvement. To enhance algorithm performances, three variants from a vertical transportation system are merged to develop sequential procedures. A vertical transportation system is the movement to the required position with or against gravity acceleration by using machine power under required conditions. Nowadays, an elevator is necessary for every multilevel building. In the current highly competitive market, elevator designs in terms of speed, capacity requirements, safety, and reliability are key components for a construction company to increase its efficiency. Most elevator producers have software to memorise the frequency of usage. With this software, the elevator will be able to identify the building levels with the high frequency usage in each period during the day. When compared to meta-heuristic methods, the parking level is the best value in each time period. From this analogy, three cases of an analysis of time and distance according to the movement of an elevator are focused and integrated to a shuffle frog leaping algorithm.

### Hybrid SFLA with type 1 motion (HSFLA1)

**Fig. 3:**
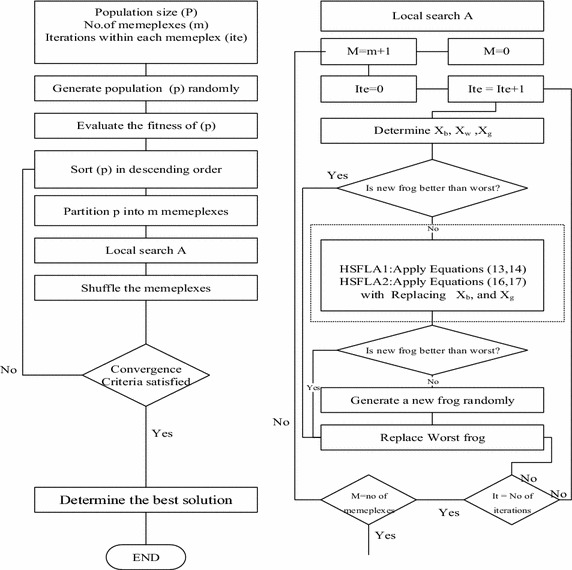
HSFLA1 and HSFLA2 flow chart

For either a type 1 motion or a motion not reaching a transitional acceleration, *S*_1_ in Eq. 13 is the movement during a constant acceleration, which can be applied to an evolutionary process of the frog group. A new position based on this motion type of the worst solution or is given by Eq. 14 and the range of the global best (*X*_*g*_) and the best solutions (*X*_*b*_) is the constant velocity of an elevator movement (Fig. 3).

13 $$S_{1} = \frac{{V_{1}^{2} }}{2a}$$

14 $$New\,position\,X_{w} = Current\,position\,X_{w} + Rand() \times S_{1}$$

### Hybrid SFLA with type 2 motion (HSFLA2)

A type 2 motion or a motion reaching a transitional acceleration is the movement in which an elevator does not reach an ending point of the transitional acceleration. A velocity *V*_2_ and corresponding distance can be represented by Eqs. 15 and 16, respectively. A new position of the worst solution or *X*_*w*_ is given by Eq. 17, where *X*_*g*_ is the global best solution and *X*_*b*_ is best solution at the current position (Fig. 3).

15 $$V_{2} = \left[ {V_{1}^{3} + 3aV_{1} \left( {\frac{{S_{T} }}{2} - S_{1} } \right)} \right]^{1/3}$$

16 $$S_{2} = \left( {\frac{1}{3a}} \right)\left( {\frac{{V_{max}^{3} }}{{V_{1} }} - V_{1}^{2} } \right) + S_{1}$$

17 $$New\,position\,X_{w} = Current\,position\,X_{w} + Rand() \times \left( {S_{2} + \left( {X_{b} - X_{g} } \right)} \right)$$

### Hybrid SFLA with type 3 motion (HSFLA3)

**Fig. 4:**
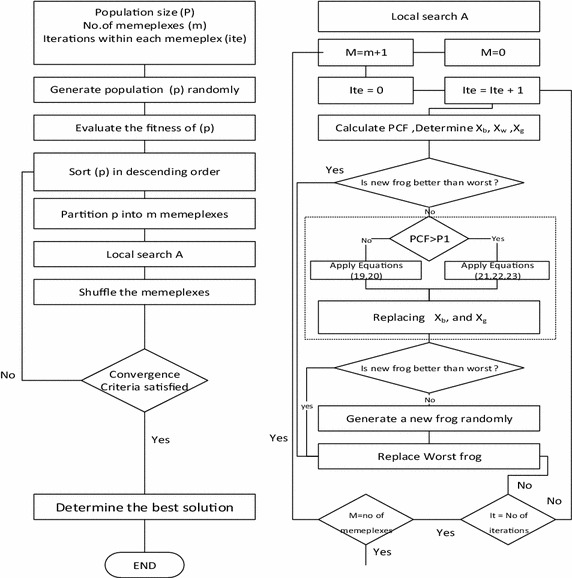
HSFLA3 flow chart

A type 3 motion occurs, when there is a circumstance having more than one command to an elevator. An actual elevator will have many types of motion. The simulation of this movement will create the probability of selection either short or long run called Probability of Choosing Floor (*PCF*). *PCF* is a simulated probability for selecting a movement type of elevator. A *PCF* value is between a minimum probability (*PCF*_*min*_) of 0.45 and a maximum probability (*PCF*_*max*_) of 0.60. Probability *P*1 is a random number between 0 and 1. If PCF values less than *P*1, short run movement will be applied. If *PCF* values more than *P*1, new position will be generated by long run movement. Leveling is an adjusting position process for protecting offset of elevator and floor. A leveling process will be applied to the last step of each actual movement of the elevator. Under the maximal iteration (*MaxIte*), a *PCF* at the current iteration (*CurIte*) can be calculated from Eq. 18. The short run movement and the new position of *X*_*w*_ will be calculated via Eqs. 19 and 20, respectively. The long run movement will be calculated via Eqs. 21 and 22. The new position of *X*_*w*_ can be calculated in Eq. 23, where $Rand \left( { - 1, 1} \right)$ is a continuous uniform random variable over (−1, 1). The flow chart of the HSFLA3 is shown in Fig. 4.

18 $$PCF = PCF_{min} + \frac{{\left( {PCF_{max} - PCF_{min} } \right) \times CurIte}}{MaxIte}$$

19 $$S_{1} = \frac{{V_{1}^{2} }}{2a}$$

20 $$New\,position\,X_{w} = Current\,position\,X_{w} + Rand\left( { - 1,1} \right) \times \left( {S_{1} } \right) + Leveling$$

21 $$S_{1} = \frac{{V_{1}^{2} }}{2a}$$

22 $$S_{2} = \left( {\frac{1}{3a}} \right)\left( {\frac{{V_{max}^{3} }}{{V_{1} }} - V_{1}^{2} } \right) + S_{1}$$

23 $$New\,position\,X_{w} = Current\,position\,X_{w} + Rand\left( { - 1,1} \right) \times \left( {S_{2} + \left( {X_{b} - X_{g} } \right)} \right) + Leveling$$

## Machining problems

### Multi-pass turning model: A

**Fig. 5:**
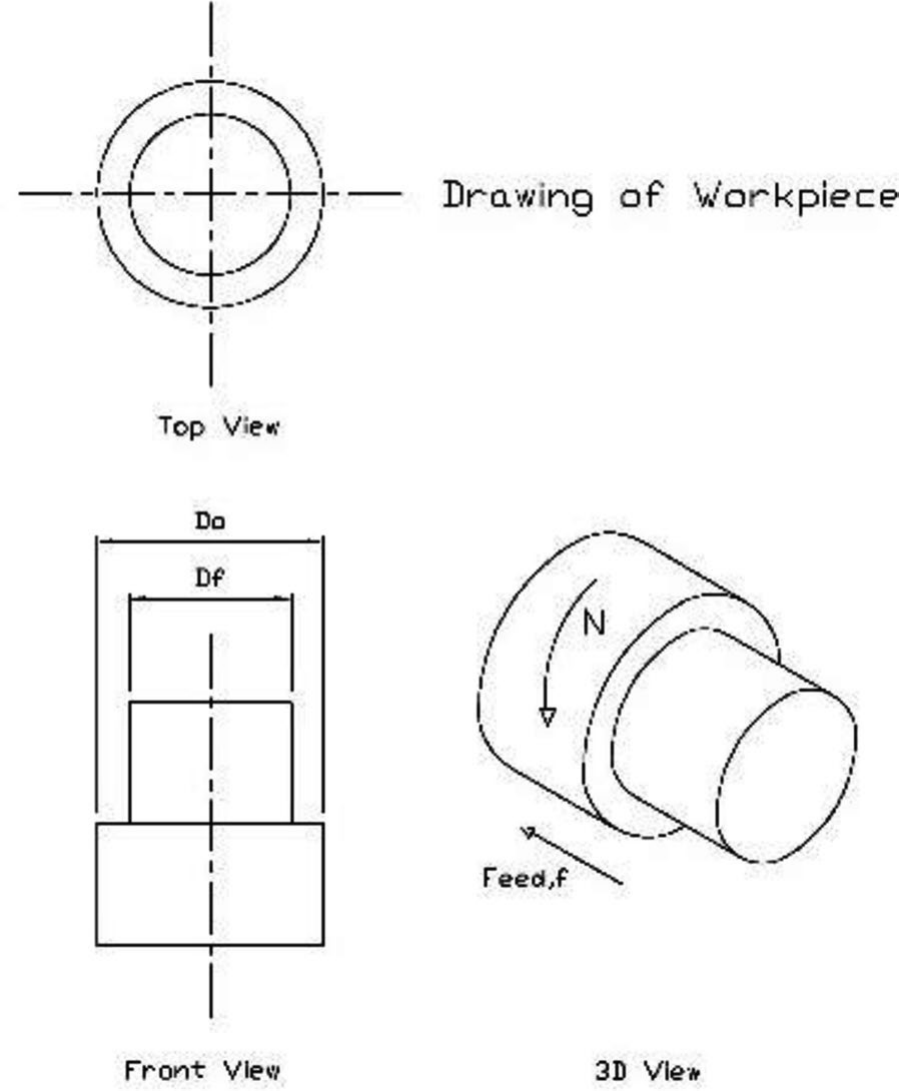
Multi-pass turning model: A

This original model was developed by Chen and Tsai. A main objective of this multi-pass turning model is to minimise a unit production cost (*C*_*U*_). *C*_*U*_ is the total cost of cutting $\left( {C_{M} } \right)$, machine idle (*C*_*I*_), tool replacement (*C*_*R*_) and tool (*C*_*T*_). The production rate is basically measured from the entire time required for producing products (*T*_*p*_). It is a function of the metal removal rate (*MRR*) and the tool life (*T*) as shown in Eq. 24. Parameters of $T_{s} , T_{c} , T_{i}$ and *V* are the tool set-up time, the tool change time, the time the tool is not cutting and the volume of the removed metal, respectively. In some operations, the parameters are set constants and *T*_*p*_ is a function of *MRR* and *T*. The *MRR* can be expressed by an analytical derivation as the product of the cutting speed, feeding and cutting depth (Eq. 25). The tool life $\left( T \right)$ is measured as the average time between the tool changes for tool sharpening. The relationship between the tool life and the parameters is defined by Taylor’s Formula (Eq. 26). All parameters of $K_{T} , \alpha_{1} , \alpha_{2}\,and\,\alpha_{3}$ are always positive. The operation cost can be expressed as the cost per product (*C*_*p*_). In the cost of the operation, two values connected with the cutting parameters (*T*, *T*_*P*_) are significant as shown in Eq. 27. Parameters of $C_{t} , C_{I}\,and\,C_{o}$ are the tool cost, the labor cost and the overhead cost, respectively. In some operations, $C_{t} , C_{I}\,and\,C_{o}$ are independent of the cutting parameters. For the cutting quality, the most important criterion for the assessment of the surface quality is roughness calculated according to Eq. 28. A specific tool-work piece combination provides the following parameters of $x_{1} , x_{2} , x_{3}\,and\,k.$ (Fig. 5).

24 $$T_{p} = T_{s} + V\frac{{\left( {1 + T_{c} /T} \right)}}{MRR} + T_{i}$$

25 $$MRR = 1000vfa$$

26 $$T = K_{T} /\left( {v^{{\alpha_{1} }} f^{{\alpha_{2} }} a^{{\alpha_{3} }} } \right)$$

27 $$C_{P} = T_{P} \left( {\frac{{C_{t} }}{T} + C_{I} + C_{O} } \right)$$

28 $$R_{a} = kv^{{x_{1} }} f^{{x_{2} }} a^{{x_{3} }}$$

One of technical specifications and organisational considerations interest is a permissible range of minimum (min) and maximum (max) of cutting conditions for the cutting speed (*v*), feed rate (*f*) and depth of cut (*a*). Due to the limitations on the machine and cutting tool and to the safety of machining, cutting parameters are limited with bottom and top permissible limits as shown in Eq. 29 There are also some implied limitations issuing from the tool characteristics and the machine capacity. For the selected tool, the tool maker identifies the limitations of the cutting conditions. The limitation on the machine is the cutting power and the cutting force (Table 1). Similarly, the machining characteristics of the work piece material are determined by physical properties. With the mechanical efficiency of the machine $\left( \eta \right)$, the consumption of the power $\left( P \right)$ can be expressed as the function of the cutting force and cutting speed (Eq. 30) and $F$ is given by Eq. 31. When Eq. 31 is introduced into Eq. 30 and $k_{n} = \frac{{k_{F} }}{{\left( {6122.45\eta } \right)}}$, Eq. 32 is obtained. The limitations of the power and cutting force are shown as Eq. 33.

**Table 1:** Parameters and description in machining model A

Parameters	Description (Unit)
*T*_*p*_	Unit machining time (min)
*π*	Mathematical constant (3.1415)
*C*_*p*_	Unit machining cost per product ($)
*R*_*a*_	Roughness of the finished surface (µm)
*MRR*	Material removal rate (mm^3^/min)
*T*_*s*_	Tool setup time (min)
*T*_*c*_	Tool change time (min)
*T*_*i*_	Tool non-cutting time (min)
*C*_*t*_	Tool cost ($)
*C*_*I*_	Labor cost ($/min)
*C*_*o*_	Overhead cost ($/min)
*K*_*F*_, *K*_*n*_, *k*, *x*_1_, *x*_2_, *x*_3_	Constants relevant to a specific tool–work piece
*K*_*T*_, *α*_1_, *α*_2_, *α*_3_, *β*_1_, *β*_2_, *β*_3_	Positive constant parameters
*V*	Volume of the removed metal (mm^3^)
*η*	Mechanical efficiency of the machine (%)
*v*_*min*_, *v*_*max*_	Boundary of cutting speed (m/min)
*f*_*min*_, *f*_*max*_	Boundary of feed rate (mm/rev)
*a*_*min*_, *a*_*max*_	Boundary of depth of cut (mm)
*F*_*max*_, *P*_*max*_	Maximum cutting force (N) and cutting power (kw)

29 $$v_{min} \le v \le v_{max} ,\quad f_{min} \le f \le f_{max} , \quad a_{min} \le a \le a_{max}$$

30 $$P = \frac{Fv}{6122.45\eta }$$

31 $$F = k_{F} f^{{\beta_{2} }} a^{{\beta_{3} }}$$

32 $$P = k_{n} f^{{\beta_{2} }} a^{{\beta_{3} }}$$

33 $$P_{{\left( {v,f,a} \right)}} \le P_{max}, \quad F_{{\left( {v,f,a} \right)}} \le F_{max}$$

Values of coefficients for A model are given later. By substituting these values, the mathematical model is derived as follows:

$$Z\left( {T_{P} , C_{P} ,R_{a} } \right) = 0.42e^{{\left( { - 0.22T_{P} } \right)}} + 0.36e^{{\left( { - 0.32C_{P} } \right)}} + 0.17e^{{\left( { - 0.26R_{a} } \right)}} + 0.05/\left( {1 + 1.22T_{P} C_{P} R_{a} } \right)$$

$$MinT_{P} = 0.12 + 231376\left( {1 + 0.26/T} \right)MRR + 0.04$$

$$MinC_{P} = \left( {13.55/T + 0.39} \right)TP$$

$$MinR_{a} = 0.0088v + 0.3232f + 0.3144a$$

Subject to:

$$T = 1575134.21\left( {v^{ - 1.7} f^{ - 1.55} a^{ - 1.22} } \right)$$

$$MRR = 1000vfa$$

$$70 \le v \le 90$$

$$0.1 \le f \le 2$$

$$0.1 \le a \le 5$$

$$0.000626\left( {vf^{1.18} a^{1.26} } \right) \le 5$$

$$1.38\left( {f^{1.18} a^{1.26} } \right) \le 230$$

### Multi-pass turning model: B

In multi-pass turning operations defined by Chen and Tsai, the objective of Eq. 34 is to minimise unit production cost (*C*_*U*_). The unit production cost includes the cutting cost (*C*_*M*_), machine idle cost (*C*_*I*_), tool replacement cost (*C*_*R*_) and tool cost (*C*_*T*_), respectively. The unit production cost (*C*_*U*_)is subject to various constraints, which are parameter bounds that cover depth of cut (Eq. 35), a cutting speed (Eq. 36) and a feed rate (Eq. 37), tool-life constraint (Eq. 38), a cutting force constraint (Eq. 39), a power constraint (Eq. 40), a stable cutting region constraint (Eq. 41), and a chip–tool interface temperature constraint (Eq. 42).

34.a $$C_{U} = C_{M} + C_{I} + C_{R} + C_{T}$$

This can be expanded as Eq. 34.b.

34.b $$C_{U} = k_{o} \left[ {\frac{\pi DL}{{1000V_{r} f_{r} }}\left( {\frac{{d_{t} - d_{s} }}{{d_{r} }}} \right) + \frac{\pi DL}{{1000V_{s} f_{s} }}} \right]\,+\,k_{o} \left[ {t_{c} + \left( {h_{1} L + h_{2} } \right)\left( {\frac{{d_{t} - d_{s} }}{{d_{r} }} + 1} \right)} \right] + k_{o} \frac{{t_{e} }}{{T_{P} }}\left[ {\frac{\pi DL}{{1000V_{r} f_{r} }}\left( {\frac{{d_{r} - d_{s} }}{{d_{r} }}} \right) + \frac{\pi DL}{{1000V_{s} f_{s} }}} \right] + \frac{{k_{t} }}{{T_{P} }}\left[ {\frac{\pi DL}{{1000V_{r} f_{r} }}\left( {\frac{{d_{r} - d_{s} }}{{d_{r} }}} \right) + \frac{\pi DL}{{1000V_{s} f_{s} }}} \right]$$

35 $$d_{rL} \le d_{r} \le d_{rU}$$

36 $$f_{rL} \le f_{r} \le f_{rU}$$

37 $$V_{rL} \le V_{r} \le V_{rU}$$

38 $$T_{L} \le T_{r} \le T_{U}$$

39 $$k_{I} f_{r}^{\mu } d_{r}^{v} \le F_{U}$$

40 $$\frac{{k_{I} f_{r}^{\mu } d_{r}^{v} V_{r} }}{6120\eta } \le P_{U}$$

41 $$V_{r}^{\lambda } f_{r} d_{r}^{v} \ge S_{c}$$

42 $$Q_{r} = k_{2} V_{r}^{\tau } f_{r}^{\phi } d_{r}^{\delta } \le Q_{U}$$

There are some surface finish machining constraints and parameter relationships. Surface finish machining constraints are depth of cut, feed rate, cutting speed, tool-life, cutting force, power, stable cutting region, chip-tool interface temperature and surface finish (Table 2). These are formulated in Eqs. (43–55), which also include the parameters relationships.

**Table 2:** Parameters and description in machining model B

Parameters	Description (Unit)
*d*_*r*_, *d*_*s*_	Depth of cut for rough and finish machining (mm)
$$d_{rL} , d_{rU}$$	Boundary of depth of cut in rough machining (mm)
$$d_{sL} , d_{sU}$$	Boundary of depth of cut in finish machining (mm)
*d*_*t*_	Depth of material to be removed (mm)
*D*, *L*	Diameter and length of work-piece (mm)
*f*_*r*_, *f*_*s*_	Feed rates in rough and finish machining (mm/rev)
$$f_{rL} , f_{rU}$$	Boundary of feed rate in rough machining (mm/rev)
$$f_{sL} , f_{sU}$$	Boundary of feed rate in finish machining (mm/rev)
*F*_*U*_	maximum cutting force (kgf)
*h*_1_, *h*_2_	Constants relating to cutting tool travel time (min)
*k*_*o*_	Labor cost include overhead cost ($/min)
*k*_*f*_	Coefficient of specific tool work-piece combination
*k*_*q*_	Coefficient of chip–tool interface temperature
*k*_*t*_	Cutting edge cost ($/edge)
$$k_{1} ,\upmu,\upupsilon$$	The constant values of cutting force equation
$$k_{2} , \tau , \phi , \delta$$	Constants related to chip-tool interface temperature equation
*k*_3_, *k*_4_, *k*_5_	Constants for roughing and finishing parameter
*n*	Integer number of rough cuts
*p*, *q*, *r*, *C*_*o*_	Constants of tool-life
$$P_{r} , P_{s}$$	Cutting power during rough and finish machining (kw)
*P*_*U*_	Maximum cutting power (kw)
$$Q_{r} , Q_{s}$$	Chip–tool interface rough and finish machining temperatures (°C)
*Q*_*U*_	Maximum allowable chip-tool interface temperature (°C)
*R*_*a*_	Maximum allowable surface roughness (mm)
*R*_*n*_	Nose radius of cutting tool (mm)
*S*_*C*_	Limit of stable cutting region constraint
*SR*_*U*_	Maximum surface roughness (mm)
*t*_*e*_	Tool exchange time (min/edge)
$$T, T_{r} , T_{s}$$	Tool life, expected tool life for rough machining and finish machining (min)
*t*_*c*_	Constant term of machine idling time (min)
*T*_*p*_	Tool life of weighted combination of *T*_*r*_ and *T*_*s*_ (min)
$$T_{U} , T_{L}$$	Boundary for tool life (min)
$$V_{r} , V_{s}$$	Cutting speeds in rough and finish machining (m/min)
*V*_*rL*_, *V*_*rU*_	Boundary of cutting speed in rough machining (m/min)
*V*_*sL*_, *V*_*sU*_	Boundary of cutting speed in finish machining (m/min)
*q*	The weight for *T*_*p*_ [0,1]
$$\lambda , \nu$$	Constants related to expression of stable cutting region
*η*	The power efficiency (%)

43 $$d_{sL} \le d_{s} \le d_{sU}$$

44 $$f_{sL} \le f_{s} \le f_{sU}$$

45 $$V_{sL} \le V_{s} \le V_{sU}$$

46 $$T_{L} \le T_{s} \le T_{U}$$

47 $$k_{I} f_{s}^{\mu } d_{s}^{v} \le F_{U}$$

48 $$P_{r} = \frac{{k_{I} f_{s}^{\mu } d_{s}^{v} V_{s} }}{6120\eta } \le P_{U}$$

49 $$V_{s}^{\lambda } f_{s} d_{s}^{v} \ge S_{c}$$

50 $$Q_{s} = k_{2} V_{s}^{r} f_{s}^{\phi } d_{s}^{\delta } \le Q_{U}$$

51 $$\frac{{f_{s}^{2} }}{{8R_{n} }} < SR_{u}$$

52 $$V_{s} \ge k_{3} V_{r}$$

53 $$f_{v} \ge k_{4} f_{s}$$

54 $$d_{r} \ge k_{5} d_{s}$$

55 $$d_{r} = \frac{{d_{t} - d_{s} }}{n}$$

In addition to these constraints, the total depth of cut is another important constraint for this model. The total depth of cut (*d*_*t*_) is the sum of the depth of the finished cut (*d*_*s*_) and the depth of the rough cut (*nd*_*r*_). The optimisation algorithm does not determine the optimal depth of roughing since it can be given by the mathematical manipulation as expressed Therefore, one can eliminate the equality constraint and the decision variable (*d*_*r*_) in the optimisation procedure:

$$d_{s} = d_{t} - nd_{r}$$

The five machining parameters $(V_{r} , f_{r} , d_{s} , V_{s} , f_{s} )$ are determined for turning model optimisation. Further details about the turning mathematical model and data with respect to machining can be obtained from Shin and Joo (1992).

## Computational results and analyses

A preliminary study used two engineering optimisation problems of single pass turning and multi-pass turning to evaluate selected approaches of original harmony search (HSA) and shuffled frog leaping (SFLA) algorithms. The first model (S) was developed for single pass turning of a medium carbon steel work piece using a carbide tool (Khan et al. 1997). The objective of this model was to minimise the production cost in dollars per piece. The problem was to evaluate the performance of various new methods and defined as follows

$${\text{min }}Cost = 452V^{ - 1} + f^{ - 1} + 10^{ - 5} V^{2.33} f^{0.4}$$

Subject to the constraints:

1. Constraint due to cutting power (*P*_*c*_): $P_{c} \le 5.5$; where, *P*_*c*_ = 10.6 × 10^−2^*Vf*^0.83^.
2. Constraint due to surface finish (*R*_*a*_): $SF \le 2\,\upmu {\text{m}}$; where, *SF* = 2.2 × 10^4^*V*^−1.52^*f*
3. The range of feed rate and cutting speed were taken as: 0 ≤ *V* ≤ 500 and 0.0 ≤ *f* ≤ 0.5

The second model (*M*), was formulated for multi-pass turning operation of a medium carbon tool. The objective was to minimise the production cost in yen per piece. Parameters of n and d_i_ are the number of passes and the depth of cut, respectively. The total depth (*A*) of the material is the sum of depths of n cuts, so $A = \sum\nolimits_{i = 1}^{n} {d_{i} }$. The problem was defined as follows:

Subject to the constraints:

1. Constraint due to cutting force (*F*_*c*_): $F_{c} \le 170 \,{\text{kg}}$; where, *F*_*c*_ = 290.73 *V*^−0.1013^*f*^0.725^*d*
2. Constraint due to stable cutting surface; *fV*^2^ ≥ 2230.5
3. Constraint due to surface roughness (*H*_*max*_): 0.356*f*^2^ ≤ *H*_*max*_
4. Constraint due to power consumption (*P*_*c*_): $P_{c} = 7.5 \,{\rm kw}$; where, $P_{c} = \frac{{F_{c} V}}{4896}$
5. The allowable ranges for these variables:

$$0 \le d \le A, 14.13 \le V \le 1005.3\, {\text{m/min}}, 0.001 \le f \le 5.6\, {\text{mm/rev}}$$

**Fig. 6:**
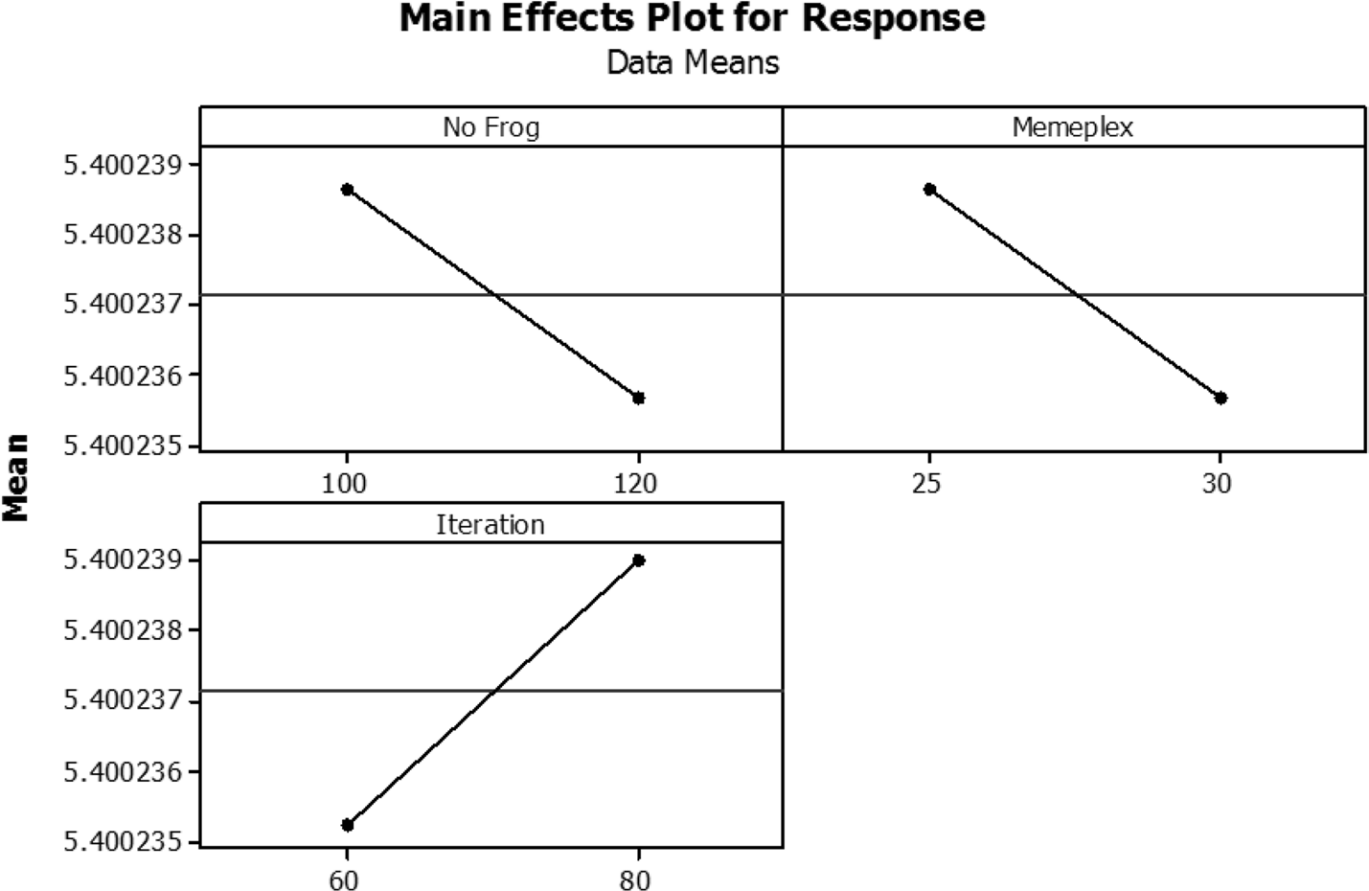
Main effect plot of the well-know Branin response function

The HSA parameters of HMS, probability of *HMCR* and probability of $PAR$ were set at 30, 0.90 and 0.35, respectively. The parameter values for SFLA on well know response functions were performed on factorial experiments are shown in Fig. 6. The preferable levels of [number of frogs (*P*), number of memeplexes (*M*), Iterations] were [100, 25, and 80]. The vertical transportation system parameters were set as: acceleration (*a*) = 1, maximal velocity (*V*_*max*_) = 0.8 and leveling = 0.005. These algorithms were executed with 6000 iterative searches (*MaxIte*). There were twenty replications in each problem. The performance of both algorithms was compared using the mean and standard deviation (STDEV) of actual process yields including the processing time to reach the optimum at the maximal preset of iterations. On the S model, the HSA seemed to be better in terms of both process yield and processing time. In the M problem, the SFLA found the better solution. In addition, the speed of convergence of the SFLA was superior in both problems (Table 3).

**Table 3:** Comparison of performance measures in a preliminary study

Model	Measures	HSA		SFLA	
		Cost (Pence)	Time (s)	Cost (Pence)	Time (s)
S	Mean	12.0981	181.4938	12.1284	220.6899
	Min	12.0980	222.0191	12.1037	280.7896
	Max	12.0985	150.6266	12.1692	182.0317
	SD	0.0002	16.74055	0.0226	29.53819

Model	Measures	HSA		SFLA	
		Cost (Yens)	Time (s)	Cost (Yens)	Time (s)
M	Mean	96.3576	211.7427	96.3525	224.368
	Min	96.0764	259.0222	96.0322	285.4694
	Max	96.4137	175.7311	96.4113	185.0655
	SD	0.0707	19.53064	0.0785	30.03049

Although the shuffle frog leaping algorithm has been used for several optimisation applications, it has not yet been reported in the literature for optimisation of machining parameters in turning operations. In this study, the proposed variants of a vertical transportation system on the SFLA were applied to machining Operation optimisation problems. On the multi-pass turning models, an important task was to find optimal cutting conditions. The turning values of coefficients were statistically determined on the basis of the data measured experimentally via tool life, roughness, manufacturing time and cutting force. Values of coefficients for the model A and B are given in Tables 4 and 5.

**Table 4:** Values of coefficients for the model A

$T_{s} = 0.12\;{ \hbox{min} }$	$T_{c} = 0.26\;{ \hbox{min} }$	$T_{i} = 0.04\;{ \hbox{min} }$
$C_{t} = 13.55 {\$}$	$C_{I} = 0.31 \$ / {\text{min}}$	$C_{o} = 0.08 \$ / {\text{min}}$
*K* = 1.001	*K*_*T*_ = 1575134.21	*K*_*F*_ = 1.38
*X*_1_ = 0.0088	*X*_2_ = 0.3232	*X*_3_ = 0.3144
*α*_1_ = 1.70	*α*_2_ = 1.55	*α*_3_ = 1.22
*β*_1_ = 0	*β*_2_ = 1.18	*β*_3_ = 1.26
$V = 231376 \;{\text{mm}}^{3}$	*η* = 36 %	$v_{min} = 70 \;{\text{m/min}}$
$v_{max} = 90\;{\text{m/min}}$	$f_{min} = 0.1\;{\text{mm/rev}}$	$f_{max} = 2\;{\text{mm/rev}}$
$a_{min} = 0.1\;{\text{mm}}$	$a_{max} = 5\;{\text{mm}}$	$F_{max} = 230 N$
$$P_{max} = 5 kw$$

**Table 5:** Values of coefficients for the model B

$D = 50\;{\text{mm}}$	$L = 300\;{\text{mm}}$	$D_{t} = 6.0\;{\text{mm}}$
$V_{rU} = 500\;{\text{m/min}}$	$V_{rL} = 50\;{\text{m/min}}$	$f_{rU} = 0.9\;{\text{mm/rev}}$
$f_{rL} = 0.1\;{\text{mm/rev}}$	$d_{rU} = 3.0\;{\text{mm}}$	$d_{rL} = 1.0\;{\text{mm}}$
$V_{sU} = 500\;{\text{m/min}}$	$V_{sL} = 50\;{\text{m/min}}$	$f_{sU} = 0.9\;{\text{mm/sev}}$
$f_{sL} = 0.1\;{\text{mm/sev}}$	$d_{sU} = 3.0\;{\text{mm}}$	$d_{sL} = 1.0\;{\text{mm}}$
$k_{o} = 0.5 \$ / {\text{min}}$	$k_{t} = 2.5 \$ / {\text{edge}}$	*h*_1_ = 7 × 10^−4^
*h*_2_ = 0.3	$t_{c} = 0.75\;{\text{min/piece}}$	$t_{e} = 1.5\;{\text{min/edge}}$
*p* = 5	*q* = 1.75	*r* = 0.75
*c*_*o*_ = 6 × 10^−11^	$T_{u} = 45\;{ \hbox{min} }$	$T_{L} = 25\;{ \hbox{min} }$
*k*_*f*_ = 108	*μ* = 0.75	*v* = 0.95
*η* = 0.85	$F_{U} = 200 \,{\text{kgf}}$	*P*_*U*_ = 5 kw
*λ* = 2	*v* = −1	*S*_*c*_ = 140
*k*_*q*_ = 132	*τ* = 0.4	*ϕ* = 0.2
*δ* = 0.105	*Q*_*u*_ = 1000 °C	$R_{n} = 1.2\;{\text{mm}}$
*k*_3_ = 1	*k*_4_ = 1	*k*_5_ = 1
*T*_*P*_ = 25	*n* = 1	*k*_1_ = 1
	*SR*_*U*_ = 10	*k*_2_ = 2.5

**Fig. 7:**
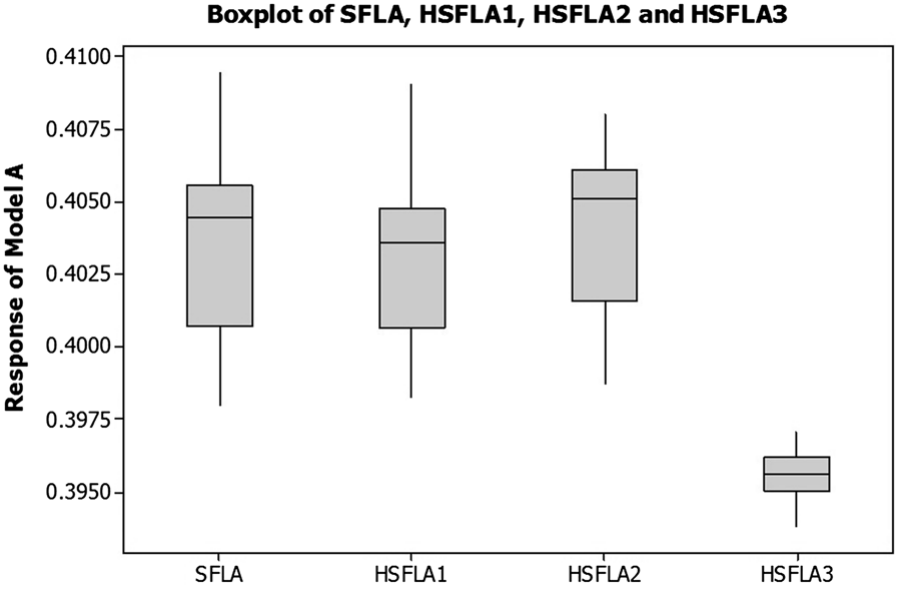
Box–Whisker graphical results on the model A

The proposed algorithms based on vertical transportation systems were programmed in a Visual C# 2008 on a Laptop ASUS A45V Series. A comparison of the conventional procedures of SFLA and three hybridisations results were presented in this section. For all meta-heuristics, their influential parameters affected algorithm performance measures such as solution quality and computational time. On the tested manufacturing problems, the experiments were run and analysed to achieve the most preferable parameter settings based on the initial levels from previous literatures. For all optimisation problems presented in this paper, parameter values for the SFLA were taken from other research, and those for evolutionary elements parameters in vertical transportation system were determined from actual elevator operations. These parameter levels were then applied throughout. The performance of the different algorithms was compared via the mean and standard deviation of actual process yields and the processing time to reach the optimum at the maximal preset of iterations. For the model A, the descriptive results found by the SFLA and all variants via a box-whisker plot are shown in Fig. 7. The HSFLA3 was statistically significant, at the 95 % confidence interval, with the lowest *T*_*P*_ value of 0.3938, when the minimum number of rough cuts or *n* of 1 was taken. Fine tuning gave solutions with two steps. The first vertical movement of *S*_2_ brought the convergence rate to near the optimal solution and the next vertical movement of *S*_1_ was used for fine turning to the optimal point. The HSFLA3 were superior in terms of sample mean, minimum and standard deviation after 500 iterations. Numerical results of the best variant (HSFLA3) from previous solutions reported in literature are in Table 6.

**Table 6:** Parameter levels from GA, TLBO and HSFLA3

Parameter	Mathematical	GA	TLBO	HSFLA3
*v* (mm/min)	86.837	86.8549	98.688	99.9494
*f* (mm/rev)	1.8601	1.8622	1.978	1.9973
*a* (mm)	4.3	4.3068	4.9449	4.9971
*T*_*P*_ (min)	0.459051	0.4938	0.4017	0.3938
*C*_*P*_ ($)	0.3114	0.3233	0.3283	0.3306
*R*_*a*_ (μm)	2.7172	2.7202	3.0624	3.0962
*MRR* (mm^3^/min)	777,820.7423	777,820.7424	965,243	997,592.7
*T* (min)	42	42.81	31.7	30.1683
*F* (*N*)	177.507	177.512	23.124	23.7029
*P* (*kw*)	0.007	0.0071	0.0868	0.0882
*Z*	0.8909	0.8861	0.8187	0.8185

**Fig. 8:**
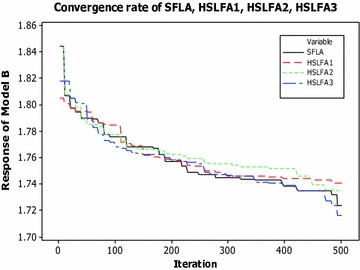
A convergence rate of the model B

The machining data required for optimal evaluation of Model B were initially analysed for different values of depth of cut without considering equality constraints on the total depth of cut (Ermer 1971). The optimal results from using the SFLA and its variants for removing a depth of 6 mm are shown in Fig. 8. From the analysis of results, HSFLA3 provided the statistically significant results, at the 95 % confidence interval, without any violation of constraints. From the analytical results for four cases a limitation of the depth of finish cut to a shorter range led to an increase in the number of passes and the optimal cost. Therefore, the same range for finish and rough cuts was introduced for removing the total depth of cut in multi-pass turning operations. An analysis of Model B was performed with an unequal constraint on total depth of cut, which only gave a limit on number of passes. Additionally, an equality constraint on total cutting depth was included on the model.

The optimal parameter levels included an optimal subdivision of depth of cut, an optimal number of passes required in each case, cutting speed and feed rates for each rough and finish pass and the optimal production cost. Analysis of results suggested that considering different ranges for finish and rough cuts was not recommended because it required more passes to remove the total depth. This resulted in an increase of total production cost. Additionally, when depths of both finish and rough cuts were in the same range, there were fewer passes with reduced production cost. Therefore, the same range for finish and rough cuts for removing the total depth of cut in multi-pass turning operations was proved to be a better choice. HSFLA3 outperformed, when compared to all other methods in the literatures. The preferable convergence rate of the HSFLA3 with 10,000 function evaluations is shown in Fig. 7. The results show that the HSFLA3 is highly competitive with other published optimisation techniques available in the literature. The HSFLA3 needs a lower number of function evaluations, improves the convergence rate, and can handle different constraint forms. The results of the SFLA and all hybridisations were compared with results of cuckoo optimisation algorithm (COA), genetic algorithms (GA), particle swarm optimisation (PSO), ant colony optimisation (ACO), hybrid particle swarm optimisation (HPSO), simulated annealing-pattern search (SA-PS), teaching–learning-based optimisation algorithm (TLBO), hybrid robust differential evolution (HRDE), artificial immune algorithm (AIA), differential evolution algorithm and receptor editing (DERE), artificial bee colony (ABC), differential evolution (DE), hybrid artificial bee colony (HABC), hybrid teaching learning based optimisation (HRTLBO), hybrid genetic algorithm sequential quadratic programming (GA-SQP), firefly (FA) and totally disturbed particle swarm optimisation (TDPSO) as shown in Table 7. The HSFLA3 obtained near optimal solution; it can be used for machining parameter selection of complex machined parts that require many machining constraints. Moreover, it can also solve the other metal cutting optimisation problems such as milling and drilling. In addition, the machining model proposed herein can be integrated into a CAD/CAM system for identifying the optimal machining parameters and reducing the manufacturing cost in metal machining.

**Table 7:** Comparison of different optimisation methods

Method	Cutting speed (m/min)		Feed rate (mm/rev)		Depth of cut (mm)		*C*_*U*_ ($/piece)	Constraint violation
	*V*_*r*_	*V*_*s*_	*f*_*r*_	*f*_*s*_	*d*_*r*_	*d*_*s*_		
COA (Mellal and Williams 2015)	123.1462	169.9876	0.5655	0.2262	3	3	1.959	–
GA (Onwubolu and Kumalo 2001)	114.22	164.369	0.7	0.2978	2.9745	2.9863	1.8450	(38), (39), (40), (46), (47), (48)
PSO (Srinivas et al. 2009)	106.69	155.89	0.897	0.28	2	2	2.272	0
ACO (Vijayakumar et al. 2003)	103.05	162.02	0.9	0.24	–	–	1.626	(55): not considered
HPSO (Costa et al. 2011)	123.3424	169.9783	0.5655	0.2262	3	3	1.959	–
SA–PS (Chen and Tsai 1996)	–	–	–	–	–	–	2.313	–
TLBO (Rao and Kalyankar 2013)	110	170	0.565	0.225	3	3	1.973	–
HRDE (Yildiz 2013a)	–	–	–	–	–	–	2.046	–
AIA (Yildiz 2013a)	–	–	–	–	–	–	2.12	–
DERE (Yildiz 2012)	–	–	–	–	–	–	2.046	–
ABC (Yildiz 2012)	–	–	–	–	–	–	2.118	–
DE (Yildiz 2012)	–	–	–	–	–	–	2.136	–
HABC (Yildiz 2013b)	–	–	–	–	–	–	2.046	–
HRTLBO (Yildiz 2013c)	–	–	–	–	–	–	2.046	–
GA–SQP (Belloufi et al. 2012)	94.464	162.289	0.866	0.258	3	3	1.814	(38), (39)
FA (Belloufi et al. 2014)	98.4102	162.2882	0.820	0.2582	3	3	1.824	(39)
TDPSO (Samuel and Rajan 2015)	123.34317	123.34317	0.565528	0.565528	3	3	1.7361	–
HSFLA3	131.7577	138.4592	0.55407	0.5056	3	3	1.7157	–

## Conclusion and future work

This paper embedded various evolutionary elements from the novel vertical transportation systems on a hybrid shuffled frog leaping algorithm. An objective is to simultaneously improve the local search stability and the global search ability for nonlinear constrained models. When optimising the machining processes is effective these parameters dramatically decrease both production cost and time and increase the final product quality. Both models of single-pass and multi-pass operations were highly constrained and nonlinear in nature. When an economic perspective under a constrictive machining environment is focused, the multi-pass operations are mainly preferred over single-pass operations. This study mainly focused on empirical models of multi-pass turning processes to determine the optimal parameter settings under the consumer production requirements in terms of better quality with lower costs. HSFLA3 outperformed on these machining optimizations, when comparing numerical results with the remaining embedded algorithms and previous studies. It may be concluded that HSFLA3 was a good choice for solving complex machining optimisation problems arising in manufacturing or other process industries. Further works include applications of the proposed methods on other turning operation models and implementations of the proposed approach to real-world problems.

## References

[CR1] Belloufi A, Assas M, Rezgui I (2012). Optimization of cutting conditions in multi-pass turning using hybrid genetic algorithm-sequential quadratic programming. J Appl Res Technol.

[CR2] Belloufi A, Assas M, Rezgui I (2014). Intelligent selection of machining parameters in multi-pass turnings using firefly algorithm. Model Simul Eng.

[CR3] Bhattacharjee KK, Sarmah SP (2014). Shuffled frog leaping algorithm and its application to 0/1 knapsack problem. Appl Soft Comput.

[CR4] Chauhan P, Pant M, Deep K (2015). Parameter optimization of multi-pass turning using chaotic PSO. Int J Mach Learn Cybernet.

[CR5] Chen M-C, Chen K-Y (2010). Optimization of multipass turning operations with genetic algorithms: a note. Int J Prod Res.

[CR6] Chen MC, Tsai DM (1996). A simulated annealing approach for optimization of multi-pass turning operations. Int J Prod Res.

[CR7] Costa A, Celano G, Fichera S (2011). Optimization of multi-pass turning economies through a hybrid particle swarm optimization technique. Int J Adv Manuf Technol.

[CR14] Dep K, Datta R (2011). Hybrid evolutionary multi-objective optimization and analysis of machining operations. Eng Optim.

[CR8] Elbeltagi E, Hegazy T, Grierson D (2007). A modified shuffled frog-leaping optimisation algorithm: applications to project management. Struct Infrastruct Eng Maint Manag Life Cycl.

[CR9] Ermer ED (1971). Optimization of the constrained machining economics problem by geometric programming. ASME J Eng Ind.

[CR10] Ermer DS, Patel DC (1974) Maximization of production rate with constraints by linear programming and sensitivity analysis. In: The 2nd North American Metalworking Research Conference (WI’74)

[CR11] Eusuff MM, Lansey KE (2003). Optimisation of water distribution network design using the shuffled frog leaping algorithm. J Water Resour Plan Manag.

[CR12] Guo J, Tang H, Sun Z, Wang S, Jia X, Chen H, Zhang Z (2015). An improved shuffled frog leaping algorithm for assembly sequence planning of remote handling maintenance in radioactive environment. Sci Technol Nucl Install.

[CR13] Jadidoleslam M, Ebrahimi A (2015). Reliability constrained generation expansion planning by a modified shuffled frog leaping algorithm. Int J Electr Power Energy Syst.

[CR15] Khan Z, Prasad LB, Singhl T (1997). Machine condition optimisation by genetic algorithms and simulated annealing. Comput Oper Res.

[CR16] Klote JH (1993). Method for calculation of elevator evacuation time. J Fire Prot Eng.

[CR17] Kumar JV, Kumar DMV (2014). Generation bidding strategy in a pool based electricity market using Shuffled Frog Leaping Algorithm. Appl Soft Comput.

[CR18] Li J, Pan Q, Xie S (2012). An effective shuffled frog-leaping algorithm for multi-objective flexible job shop scheduling problems. Appl Math Comput.

[CR19] Lu K, Jing M, Zhang X, Liu H (2013). Optimization of sequential subdivision of depth of cut in turning operations using dynamic programming. Int J Adv Manuf Technol.

[CR20] Luo J, Chen M-R (2014). Improved shuffled frog leaping algorithm and its multi-phase model for multi-depot vehicle routing problem. Expert Syst Appl.

[CR21] Mellal MA, Williams EJ (2015). Cuckoo optimization algorithm for unit production cost in multi-pass turning operations. Int J Adv Manuf Technol.

[CR22] Niknam T, Mr Narimani, Jabbari M, Malekpour AR (2011). A modified shuffle frog leaping algorithm for multi-objective optimal power flow. Energy.

[CR23] Onwubolu GC, Kumalo T (2001). Optimization of multipass turning operations with genetic algorithms. Int J Prod Res.

[CR24] Rao RV, Kalyankar V (2013). Multi-pass turning process parameter optimization using teaching-learning-based optimization algorithm. Scientia Iranica.

[CR25] Roy P (2011) A new technique to solve minimum spanning tree (MST) problem using modified shuffled frog-leaping algorithm (MSFLA) with GA cross-over. In: Paper presented at the 3rd International Conference on Advances in Recent Technologies in Communication and Computing (ARTCom 2011), Bangalore

[CR26] Samuel GG, Rajan CCA (2015). Hybrid: particle swarm optimisation-genetic algorithm and particle swarm optimisation-shuffled frog leaping algorithm for long-term generator maintenance scheduling. Electr Power Energy Syst.

[CR27] Shang YL, Bouffanais R (2014). Influence of the number of topologically interacting neighbors on swarm dynamics. Sci Rep.

[CR28] Sharma S, Sharma TK, Pant M, Rajpurohit J, Naruka B (2015) Accelerated shuffled frog-leaping algorithm. In: Fourth international conference on soft computing for problem solving. Springer, India

[CR29] Shin YC, Joo YS (1992). Optimization of machining conditions with practical constraints. Int J Prod Res.

[CR30] Srinivas J, Giri R, Yang S (2009). Optimization of multi-pass turning using particle swarm intelligence. Int J Adv Manuf Technol.

[CR31] Strakosch GR, Caporale RS (2010). The vertical transportation handbook.

[CR32] Vijayakumar K, Kumudinidevi DRP (2007). A new method for optimal location of facts controllers using genetic algorithm. J Theor Appl Inf Technol.

[CR33] Vijayakumar K, Prabhaharan G, Asokan P, Saravanan R (2003). Optimization of multi-pass turning operation using ant colony system. Int J Mach Tools Manuf.

[CR34] Wang Y-C (2007). A note on optimization of multi-pass turning operations using ant colony system. Int J Mach Tools Manuf.

[CR35] Yammani C (2011) Optimal placement and sizing of the DER in distribution systems using shuffled frog leaping algorithm. In: Paper presented at the recent advances in intelligent computational systems (RAICS) IEEE, Trivandrum

[CR36] Yildiz A (2012). A comparative study of population-based optimisation algorithms for turning operations. Inf Sci.

[CR37] Yildiz A (2013). Hybrid Taguchi differential evolution algorithm for optimization of multi-pass turning operations. Appl Soft Comput.

[CR38] Yildiz A (2013). Optimization of cutting parameters in multi-pass turning using artificial bee colony-based approach. Inf Sci.

[CR39] Yildiz A (2013). Optimization of multi-pass turning operations using hybrid teaching learning based approach. Int J Adv Manuf Technol.

[CR40] Yildiz AR (2013). A new hybrid differential evolution algorithm for the selection of optimal machining parameters in milling operations. Appl Soft Comput.

[CR41] Yildiz A, Ozturk F (2006). Hybrid enhanced genetic algorithm to select optimal machining parameters in turning operation. Proc Inst Mech Eng D J Automob Eng.

[CR42] Yusup N, Zain AM, Hashim SZM (2012). Evolutionary techniques in optimizing machining parameters. Expert Syst Appl Int J.

[CR43] Zarei O, Fesanghary M, Farshi B, Saffar RJ, Razfar MR (2009). Optimization of multi-pass face-milling via harmony search algorithm. J Mater Process Technol.

[CR44] Zhang X, Zhang Y, Shi Y, Zhao L, Zoua C (2012). Power control algorithm in cognitive radio system based on modified shuffled frog leaping algorithm. Int J Electron Commun.

[CR45] Zhu GY, Zhang WB (2014). An improved shuffled frog-leaping algorithm to optimise component pick-and-place sequencing optimisation problem. Expert Syst Appl Int J.

